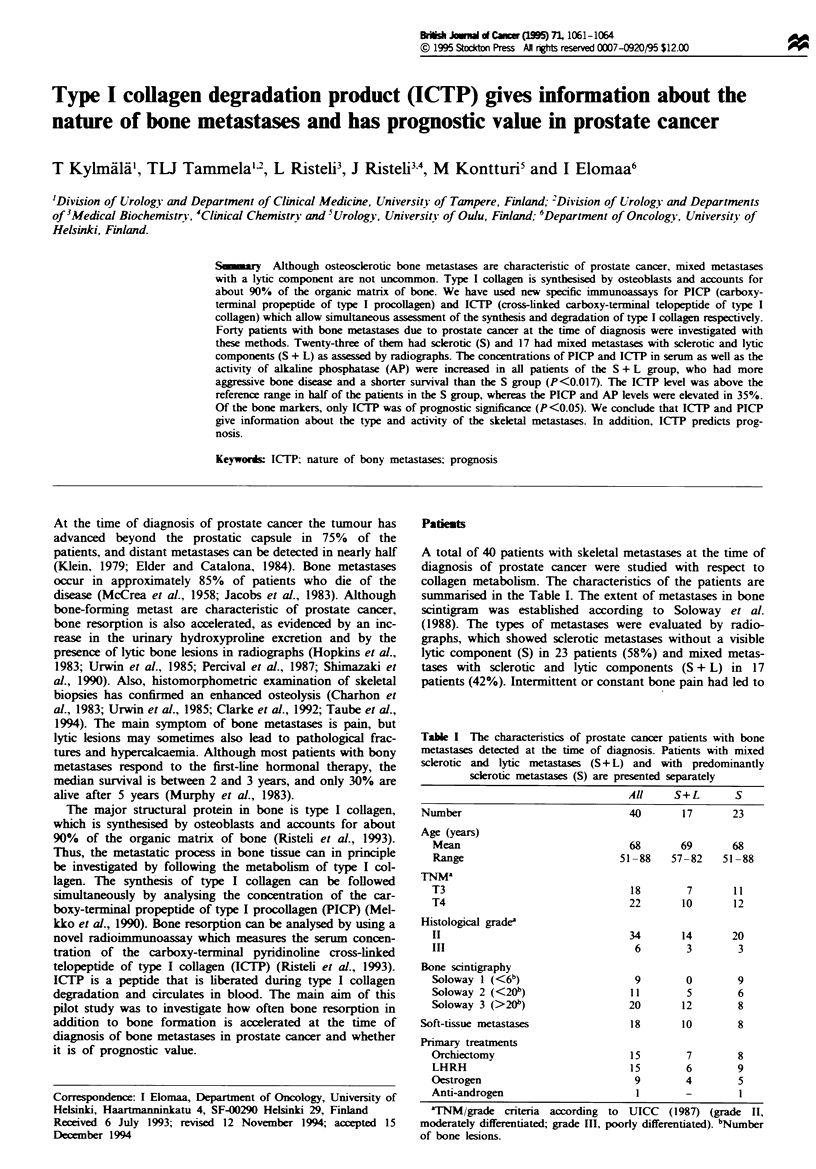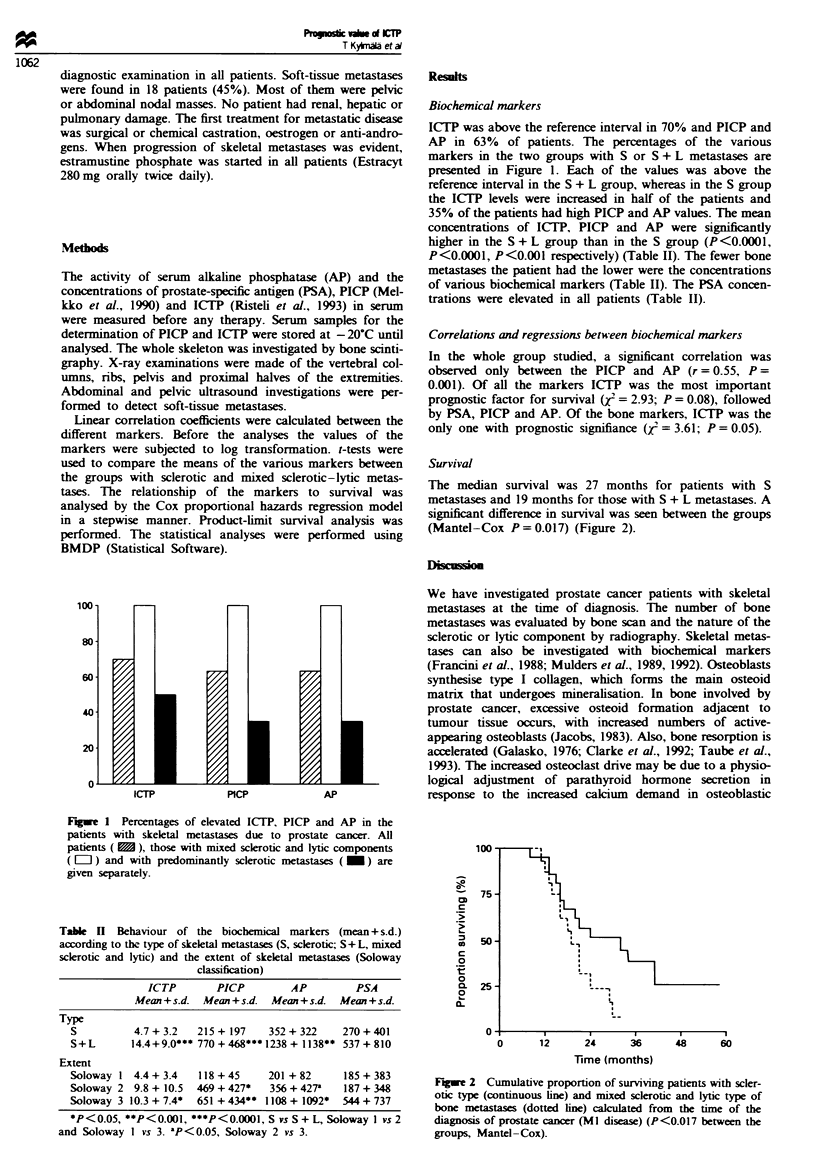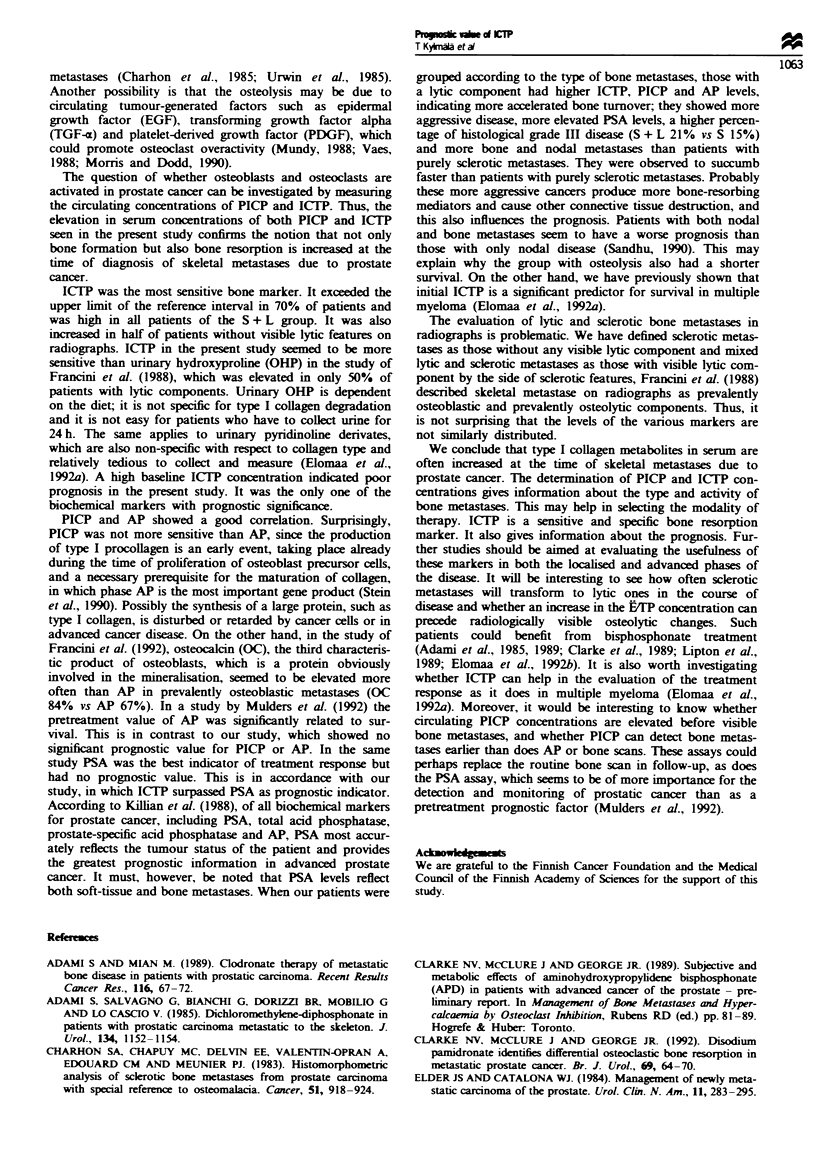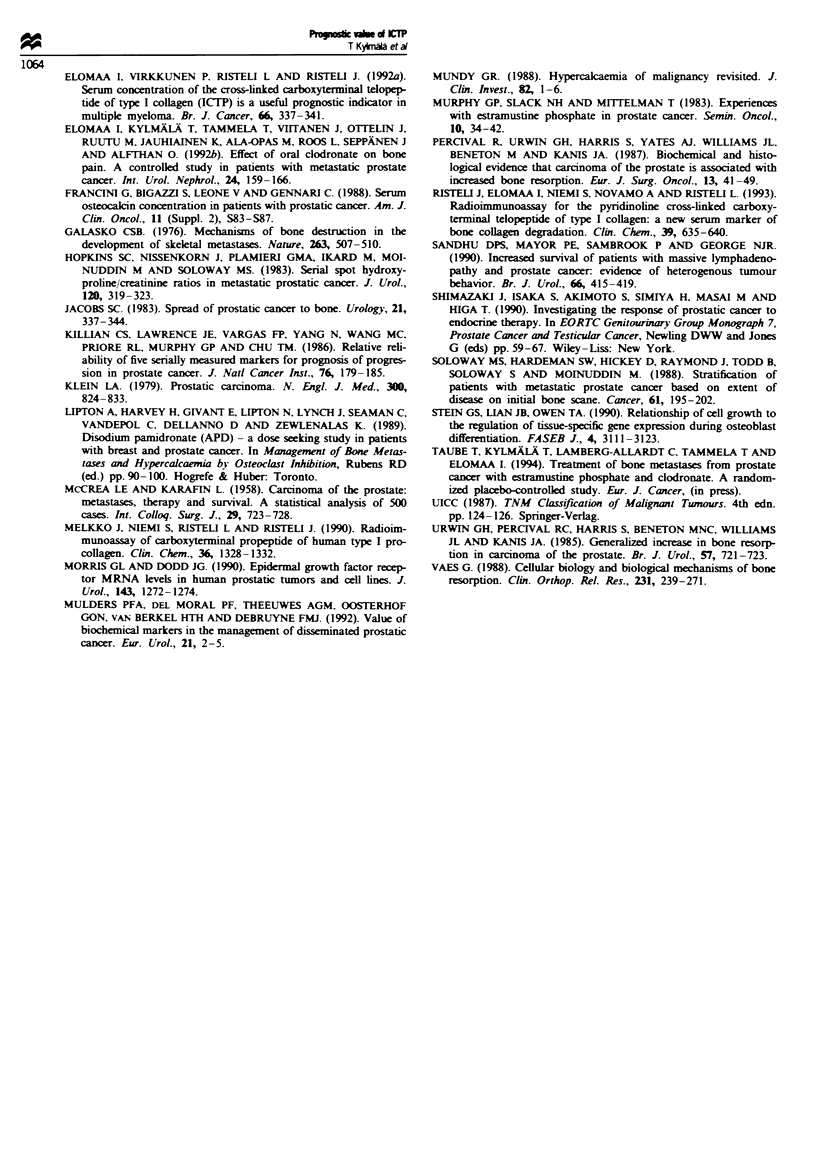# Type I collagen degradation product (ICTP) gives information about the nature of bone metastases and has prognostic value in prostate cancer.

**DOI:** 10.1038/bjc.1995.204

**Published:** 1995-05

**Authors:** T. Kylmälä, T. L. Tammela, L. Risteli, J. Risteli, M. Kontturi, I. Elomaa

**Affiliations:** Division of Urology, University of Tampere, Finland.

## Abstract

Although osteosclerotic bone metastases are characteristic of prostate cancer, mixed metastases with a lytic component are not uncommon. Type I collagen is synthesised by osteoblasts and accounts for about 90% of the organic matrix of bone. We have used new specific immunoassays for PICP (carboxy-terminal propeptide of type I procollagen) and ICTP (cross-linked carboxy-terminal telopeptide of type I collagen) which allow simultaneous assessment of the synthesis and degradation of type I collagen respectively. Forty patients with bone metastases due to prostate cancer at the time of diagnosis were investigated with these methods. Twenty-three of them had sclerotic (S) and 17 had mixed metastases with sclerotic and lytic components (S + L) as assessed by radiographs. The concentrations of PICP and ICTP in serum as well as the activity of alkaline phosphatase (AP) were increased in all patients of the S + L group, who had more aggressive bone disease and a shorter survival than the S group (P < 0.017). The ICTP level was above the reference range in half of the patients in the S group, whereas the PICP and AP levels were elevated in 35%. Of the bone markers, only ICTP was of prognostic significance (P < .05). We conclude that ICTP and PICP give information about the type and activity of the skeletal metastases. In addition, ICTP predicts prognosis.


British Journal of Cancer (1995) 71, 1061-1064

© 1995 Stockton Press All rights reserved 0007-0920/95 $12.00

### Type I collagen degradation product (ICTP) gives information about the nature of bone metastases and has prognostic value in prostate cancer

T Kylmälä[1], TLJ Tammela[1,2], L Risteli[3], J Risteli[3,4], M Kontturi[5] and I Elomaa[6]

[1]*Division of Urology and Department of Clinical Medicine, University of Tampere, Finland;* [2]*Division of Urology and Departments of* [3]*Medical Biochemistry,* [4]*Clinical Chemistry and* [5]*Urology, University of Oulu, Finland;* [6]*Department of Oncology, University of Helsinki, Finland.*

**Summary** Although osteosclerotic bone metastases are characteristic of prostate cancer, mixed metastases with a lytic component are not uncommon. Type I collagen is synthesised by osteoblasts and accounts for about 90% of the organic matrix of bone. We have used new specific immunoassays for PICP (carboxy-terminal propeptide of type I procollagen) and ICTP (cross-linked carboxy-terminal telopeptide of type I collagen) which allow simultaneous assessment of the synthesis and degradation of type I collagen respectively. Forty patients with bone metastases due to prostate cancer at the time of diagnosis were investigated with these methods. Twenty-three of them had sclerotic (S) and 17 had mixed metastases with sclerotic and lytic components (S + L) as assessed by radiographs. The concentrations of PICP and ICTP in serum as well as the activity of alkaline phosphatase (AP) were increased in all patients of the S + L group, who had more aggressive bone disease and a shorter survival than the S group ($P<0.017$). The ICTP level was above the reference range in half of the patients in the S group, whereas the PICP and AP levels were elevated in 35%. Of the bone markers, only ICTP was of prognostic significance ($P<0.05$). We conclude that ICTP and PICP give information about the type and activity of the skeletal metastases. In addition, ICTP predicts prognosis.

**Keywords:** ICTP; nature of bony metastases; prognosis

At the time of diagnosis of prostate cancer the tumour has advanced beyond the prostatic capsule in 75% of the patients, and distant metastases can be detected in nearly half (Klein, 1979; Elder and Catalona, 1984). Bone metastases occur in approximately 85% of patients who die of the disease (McCrea *et al.*, 1958; Jacobs *et al.*, 1983). Although bone-forming metast are characteristic of prostate cancer, bone resorption is also accelerated, as evidenced by an increase in the urinary hydroxyproline excretion and by the presence of lytic bone lesions in radiographs (Hopkins *et al.*, 1983; Urwin *et al.*, 1985; Percival *et al.*, 1987; Shimazaki *et al.*, 1990). Also, histomorphometric examination of skeletal biopsies has confirmed an enhanced osteolysis (Charhon *et al.*, 1983; Urwin *et al.*, 1985; Clarke *et al.*, 1992; Taube *et al.*, 1994). The main symptom of bone metastases is pain, but lytic lesions may sometimes also lead to pathological fractures and hypercalcaemia. Although most patients with bony metastases respond to the first-line hormonal therapy, the median survival is between 2 and 3 years, and only 30% are alive after 5 years (Murphy *et al.*, 1983).

The major structural protein in bone is type I collagen, which is synthesised by osteoblasts and accounts for about 90% of the organic matrix of bone (Risteli *et al.*, 1993). Thus, the metastatic process in bone tissue can in principle be investigated by following the metabolism of type I collagen. The synthesis of type I collagen can be followed simultaneously by analysing the concentration of the carboxy-terminal propeptide of type I procollagen (PICP) (Melkko *et al.*, 1990). Bone resorption can be analysed by using a novel radioimmunoassay which measures the serum concentration of the carboxy-terminal pyridinoline cross-linked telopeptide of type I collagen (ICTP) (Risteli *et al.*, 1993). ICTP is a peptide that is liberated during type I collagen degradation and circulates in blood. The main aim of this pilot study was to investigate how often bone resorption in addition to bone formation is accelerated at the time of diagnosis of bone metastases in prostate cancer and whether it is of prognostic value.

Correspondence: I Elomaa, Department of Oncology, University of Helsinki, Haartmanninkatu 4, SF-00290 Helsinki 29, Finland
Received 6 July 1993; revised 12 November 1994; accepted 15 December 1994

### Patients

A total of 40 patients with skeletal metastases at the time of diagnosis of prostate cancer were studied with respect to collagen metabolism. The characteristics of the patients are summarised in the Table I. The extent of metastases in bone scintigram was established according to Soloway *et al.* (1988). The types of metastases were evaluated by radiographs, which showed sclerotic metastases without a visible lytic component (S) in 23 patients (58%) and mixed metastases with sclerotic and lytic components (S + L) in 17 patients (42%). Intermittent or constant bone pain had led to

**Table I** The characteristics of prostate cancer patients with bone metastases detected at the time of diagnosis. Patients with mixed sclerotic and lytic metastases (S+L) and with predominantly sclerotic metastases (S) are presented separately

| | *All* | *S+L* | *S* |
|---|---|---|---|
| Number | 40 | 17 | 23 |
| Age (years) | | | |
| Mean | 68 | 69 | 68 |
| Range | 51-88 | 57-82 | 51-88 |
| TNM[a] | | | |
| T3 | 18 | 7 | 11 |
| T4 | 22 | 10 | 12 |
| Histological grade[a] | | | |
| II | 34 | 14 | 20 |
| III | 6 | 3 | 3 |
| Bone scintigraphy | | | |
| Soloway 1 (<6[b]) | 9 | 0 | 9 |
| Soloway 2 (<20[b]) | 11 | 5 | 6 |
| Soloway 3 (>20[b]) | 20 | 12 | 8 |
| Soft-tissue metastases | 18 | 10 | 8 |
| Primary treatments | | | |
| Orchiectomy | 15 | 7 | 8 |
| LHRH | 15 | 6 | 9 |
| Oestrogen | 9 | 4 | 5 |
| Anti-androgen | 1 | – | 1 |

[a]TNM/grade criteria according to UICC (1987) (grade II, moderately differentiated; grade III, poorly differentiated). [b]Number of bone lesions.

diagnostic examination in all patients. Soft-tissue metastases were found in 18 patients (45%). Most of them were pelvic or abdominal nodal masses. No patient had renal, hepatic or pulmonary damage. The first treatment for metastatic disease was surgical or chemical castration, oestrogen or anti-androgens. When progression of skeletal metastases was evident, estramustine phosphate was started in all patients (Estracyt 280 mg orally twice daily).

### Methods

The activity of serum alkaline phosphatase (AP) and the concentrations of prostate-specific antigen (PSA), PICP (Melkko *et al.*, 1990) and ICTP (Risteli *et al.*, 1993) in serum were measured before any therapy. Serum samples for the determination of PICP and ICTP were stored at −20°C until analysed. The whole skeleton was investigated by bone scintigraphy. X-ray examinations were made of the vertebral columns, ribs, pelvis and proximal halves of the extremities. Abdominal and pelvic ultrasound investigations were performed to detect soft-tissue metastases.

Linear correlation coefficients were calculated between the different markers. Before the analyses the values of the markers were subjected to log transformation. *t*-tests were used to compare the means of the various markers between the groups with sclerotic and mixed sclerotic–lytic metastases. The relationship of the markers to survival was analysed by the Cox proportional hazards regression model in a stepwise manner. Product-limit survival analysis was performed. The statistical analyses were performed using BMDP (Statistical Software).

**Figure 1** Percentages of elevated ICTP, PICP and AP in the patients with skeletal metastases due to prostate cancer. All patients ( ▨ ), those with mixed sclerotic and lytic components ( □ ) and with predominantly sclerotic metastases ( ■ ) are given separately.

**Table II** Behaviour of the biochemical markers (mean + s.d.) according to the type of skeletal metastases (S, sclerotic; S + L, mixed sclerotic and lytic) and the extent of skeletal metastases (Soloway classification)

| | *ICTP* *Mean + s.d.* | *PICP* *Mean + s.d.* | *AP* *Mean + s.d.* | *PSA* *Mean + s.d.* |
|---|---|---|---|---|
| Type | | | | |
| S | 4.7 + 3.2 | 215 + 197 | 352 + 322 | 270 + 401 |
| S + L | 14.4 + 9.0*** | 770 + 468*** | 1238 + 1138** | 537 + 810 |
| Extent | | | | |
| Soloway 1 | 4.4 + 3.4 | 118 + 45 | 201 + 82 | 185 + 383 |
| Soloway 2 | 9.8 + 10.5 | 469 + 427* | 356 + 427[a] | 187 + 348 |
| Soloway 3 | 10.3 + 7.4* | 651 + 434** | 1108 + 1092* | 544 + 737 |

*$P<0.05$, **$P<0.001$, ***$P<0.0001$, S *vs* S + L, Soloway 1 *vs* 2 and Soloway 1 *vs* 3. [a]$P<0.05$, Soloway 2 *vs* 3.

### Results

### *Biochemical markers*

ICTP was above the reference interval in 70% and PICP and AP in 63% of patients. The percentages of the various markers in the two groups with S or S + L metastases are presented in Figure 1. Each of the values was above the reference interval in the S + L group, whereas in the S group the ICTP levels were increased in half of the patients and 35% of the patients had high PICP and AP values. The mean concentrations of ICTP, PICP and AP were significantly higher in the S + L group than in the S group ($P<0.0001$, $P<0.0001$, $P<0.001$ respectively) (Table II). The fewer bone metastases the patient had the lower were the concentrations of various biochemical markers (Table II). The PSA concentrations were elevated in all patients (Table II).

### *Correlations and regressions between biochemical markers*

In the whole group studied, a significant correlation was observed only between the PICP and AP ($r = 0.55$, $P = 0.001$). Of all the markers ICTP was the most important prognostic factor for survival ($\chi^2 = 2.93$; $P = 0.08$), followed by PSA, PICP and AP. Of the bone markers, ICTP was the only one with prognostic signifiance ($\chi^2 = 3.61$; $P = 0.05$).

### *Survival*

The median survival was 27 months for patients with S metastases and 19 months for those with S + L metastases. A significant difference in survival was seen between the groups (Mantel–Cox $P = 0.017$) (Figure 2).

### Discussion

We have investigated prostate cancer patients with skeletal metastases at the time of diagnosis. The number of bone metastases was evaluated by bone scan and the nature of the sclerotic or lytic component by radiography. Skeletal metastases can also be investigated with biochemical markers (Francini *et al.*, 1988; Mulders *et al.*, 1989, 1992). Osteoblasts synthesise type I collagen, which forms the main osteoid matrix that undergoes mineralisation. In bone involved by prostate cancer, excessive osteoid formation adjacent to tumour tissue occurs, with increased numbers of active-appearing osteoblasts (Jacobs, 1983). Also, bone resorption is accelerated (Galasko, 1976; Clarke *et al.*, 1992; Taube *et al.*, 1993). The increased osteoclast drive may be due to a physiological adjustment of parathyroid hormone secretion in response to the increased calcium demand in osteoblastic

**Figure 2** Cumulative proportion of surviving patients with sclerotic type (continuous line) and mixed sclerotic and lytic type of bone metastases (dotted line) calculated from the time of the diagnosis of prostate cancer (M1 disease) ($P<0.017$ between the groups, Mantel–Cox).

metastases (Charhon et al., 1985; Urwin et al., 1985). Another possibility is that the osteolysis may be due to circulating tumour-generated factors such as epidermal growth factor (EGF), transforming growth factor alpha (TGF-α) and platelet-derived growth factor (PDGF), which could promote osteoclast overactivity (Mundy, 1988; Vaes, 1988; Morris and Dodd, 1990).

The question of whether osteoblasts and osteoclasts are activated in prostate cancer can be investigated by measuring the circulating concentrations of PICP and ICTP. Thus, the elevation in serum concentrations of both PICP and ICTP seen in the present study confirms the notion that not only bone formation but also bone resorption is increased at the time of diagnosis of skeletal metastases due to prostate cancer.

ICTP was the most sensitive bone marker. It exceeded the upper limit of the reference interval in 70% of patients and was high in all patients of the S + L group. It was also increased in half of patients without visible lytic features on radiographs. ICTP in the present study seemed to be more sensitive than urinary hydroxyproline (OHP) in the study of Francini et al. (1988), which was elevated in only 50% of patients with lytic components. Urinary OHP is dependent on the diet; it is not specific for type I collagen degradation and it is not easy for patients who have to collect urine for 24 h. The same applies to urinary pyridinoline derivates, which are also non-specific with respect to collagen type and relatively tedious to collect and measure (Elomaa et al., 1992a). A high baseline ICTP concentration indicated poor prognosis in the present study. It was the only one of the biochemical markers with prognostic significance.

PICP and AP showed a good correlation. Surprisingly, PICP was not more sensitive than AP, since the production of type I procollagen is an early event, taking place already during the time of proliferation of osteoblast precursor cells, and a necessary prerequisite for the maturation of collagen, in which phase AP is the most important gene product (Stein et al., 1990). Possibly the synthesis of a large protein, such as type I collagen, is disturbed or retarded by cancer cells or in advanced cancer disease. On the other hand, in the study of Francini et al. (1992), osteocalcin (OC), the third characteristic product of osteoblasts, which is a protein obviously involved in the mineralisation, seemed to be elevated more often than AP in prevalently osteoblastic metastases (OC 84% vs AP 67%). In a study by Mulders et al. (1992) the pretreatment value of AP was significantly related to survival. This is in contrast to our study, which showed no significant prognostic value for PICP or AP. In the same study PSA was the best indicator of treatment response but had no prognostic value. This is in accordance with our study, in which ICTP surpassed PSA as prognostic indicator. According to Killian et al. (1988), of all biochemical markers for prostate cancer, including PSA, total acid phosphatase, prostate-specific acid phosphatase and AP, PSA most accurately reflects the tumour status of the patient and provides the greatest prognostic information in advanced prostate cancer. It must, however, be noted that PSA levels reflect both soft-tissue and bone metastases. When our patients were grouped according to the type of bone metastases, those with a lytic component had higher ICTP, PICP and AP levels, indicating more accelerated bone turnover; they showed more aggressive disease, more elevated PSA levels, a higher percentage of histological grade III disease (S + L 21% vs S 15%) and more bone and nodal metastases than patients with purely sclerotic metastases. They were observed to succumb faster than patients with purely sclerotic metastases. Probably these more aggressive cancers produce more bone-resorbing mediators and cause other connective tissue destruction, and this also influences the prognosis. Patients with both nodal and bone metastases seem to have a worse prognosis than those with only nodal disease (Sandhu, 1990). This may explain why the group with osteolysis also had a shorter survival. On the other hand, we have previously shown that initial ICTP is a significant predictor for survival in multiple myeloma (Elomaa et al., 1992a).

The evaluation of lytic and sclerotic bone metastases in radiographs is problematic. We have defined sclerotic metastases as those without any visible lytic component and mixed lytic and sclerotic metastases as those with visible lytic component by the side of sclerotic features, Francini et al. (1988) described skeletal metastase on radiographs as prevalently osteoblastic and prevalently osteolytic components. Thus, it is not surprising that the levels of the various markers are not similarly distributed.

We conclude that type I collagen metabolites in serum are often increased at the time of skeletal metastases due to prostate cancer. The determination of PICP and ICTP concentrations gives information about the type and activity of bone metastases. This may help in selecting the modality of therapy. ICTP is a sensitive and specific bone resorption marker. It also gives information about the prognosis. Further studies should be aimed at evaluating the usefulness of these markers in both the localised and advanced phases of the disease. It will be interesting to see how often sclerotic metastases will transform to lytic ones in the course of disease and whether an increase in the ËTP concentration can precede radiologically visible osteolytic changes. Such patients could benefit from bisphosphonate treatment (Adami et al., 1985, 1989; Clarke et al., 1989; Lipton et al., 1989; Elomaa et al., 1992b). It is also worth investigating whether ICTP can help in the evaluation of the treatment response as it does in multiple myeloma (Elomaa et al., 1992a). Moreover, it would be interesting to know whether circulating PICP concentrations are elevated before visible bone metastases, and whether PICP can detect bone metastases earlier than does AP or bone scans. These assays could perhaps replace the routine bone scan in follow-up, as does the PSA assay, which seems to be of more importance for the detection and monitoring of prostatic cancer than as a pretreatment prognostic factor (Mulders et al., 1992).

**Acknowledgements**
We are grateful to the Finnish Cancer Foundation and the Medical Council of the Finnish Academy of Sciences for the support of this study.

**References**

ADAMI S AND MIAN M. (1989). Clodronate therapy of metastatic bone disease in patients with prostatic carcinoma. *Recent Results Cancer Res.*, **116**, 67-72.

ADAMI S, SALVAGNO G, BIANCHI G, DORIZZI BR, MOBILIO G AND LO CASCIO V. (1985). Dichloromethylene-diphosphonate in patients with prostatic carcinoma metastatic to the skeleton. *J. Urol.*, **134**, 1152-1154.

CHARHON SA, CHAPUY MC, DELVIN EE, VALENTIN-OPRAN A, EDOUARD CM AND MEUNIER PJ. (1983). Histomorphometric analysis of sclerotic bone metastases from prostate carcinoma with special reference to osteomalacia. *Cancer*, **51**, 918-924.

CLARKE NV, MCCLURE J AND GEORGE JR. (1989). Subjective and metabolic effects of aminohydroxypropylidene bisphosphonate (APD) in patients with advanced cancer of the prostate – preliminary report. In *Management of Bone Metastases and Hypercalcaemia by Osteoclast Inhibition*, Rubens RD (ed.) pp. 81-89. Hogrefe & Huber: Toronto.

CLARKE NV, MCCLURE J AND GEORGE JR. (1992). Disodium pamidronate identifies differential osteoclastic bone resorption in metastatic prostate cancer. *Br. J. Urol.*, **69**, 64-70.

ELDER JS AND CATALONA WJ. (1984). Management of newly metastatic carcinoma of the prostate. *Urol. Clin. N. Am.*, **11**, 283-295.

ELOMAA I, VIRKKUNEN P, RISTELI L AND RISTELI J. (1992*a*). Serum concentration of the cross-linked carboxyterminal telopeptide of type I collagen (ICTP) is a useful prognostic indicator in multiple myeloma. *Br. J. Cancer*, **66**, 337–341.

ELOMAA I, KYLMÄLÄ T, TAMMELA T, VIITANEN J, OTTELIN J, RUUTU M, JAUHIAINEN K, ALA-OPAS M, ROOS L, SEPPÄNEN J AND ALFTHAN O. (1992*b*). Effect of oral clodronate on bone pain. A controlled study in patients with metastatic prostate cancer. *Int. Urol. Nephrol.*, **24**, 159–166.

FRANCINI G, BIGAZZI S, LEONE V AND GENNARI C. (1988). Serum osteocalcin concentration in patients with prostatic cancer. *Am. J. Clin. Oncol.*, **11** (Suppl. 2), S83–S87.

GALASKO CSB. (1976). Mechanisms of bone destruction in the development of skeletal metastases. *Nature*, **263**, 507–510.

HOPKINS SC, NISSENKORN J, PLAMIERI GMA, IKARD M, MOINUDDIN M AND SOLOWAY MS. (1983). Serial spot hydroxyproline/creatinine ratios in metastatic prostatic cancer. *J. Urol.*, **120**, 319–323.

JACOBS SC. (1983). Spread of prostatic cancer to bone. *Urology*, **21**, 337–344.

KILLIAN CS, LAWRENCE JE, VARGAS FP, YANG N, WANG MC, PRIORE RL, MURPHY GP AND CHU TM. (1986). Relative reliability of five serially measured markers for prognosis of progression in prostate cancer. *J. Natl Cancer Inst.*, **76**, 179–185.

KLEIN LA. (1979). Prostatic carcinoma. *N. Engl. J. Med.*, **300**, 824–833.

LIPTON A, HARVEY H, GIVANT E, LIPTON N, LYNCH J, SEAMAN C, VANDEPOL C, DELLANNO D AND ZEWLENALAS K. (1989). Disodium pamidronate (APD) – a dose seeking study in patients with breast and prostate cancer. In *Management of Bone Metastases and Hypercalcaemia by Osteoclast Inhibition*, Rubens RD (ed.) pp. 90–100. Hogrefe & Huber: Toronto.

MCCREA LE AND KARAFIN L. (1958). Carcinoma of the prostate: metastases, therapy and survival. A statistical analysis of 500 cases. *Int. Colloq. Surg. J.*, **29**, 723–728.

MELKKO J, NIEMI S, RISTELI L AND RISTELI J. (1990). Radioimmunoassay of carboxyterminal propeptide of human type I procollagen. *Clin. Chem.*, **36**, 1328–1332.

MORRIS GL AND DODD JG. (1990). Epidermal growth factor receptor MRNA levels in human prostatic tumors and cell lines. *J. Urol.*, **143**, 1272–1274.

MULDERS PFA, DEL MORAL PF, THEEUWES AGM, OOSTERHOF GON, VAN BERKEL HTH AND DEBRUYNE FMJ. (1992). Value of biochemical markers in the management of disseminated prostatic cancer. *Eur. Urol.*, **21**, 2–5.

MUNDY GR. (1988). Hypercalcaemia of malignancy revisited. *J. Clin. Invest.*, **82**, 1–6.

MURPHY GP, SLACK NH AND MITTELMAN T (1983). Experiences with estramustine phosphate in prostate cancer. *Semin. Oncol.*, **10**, 34–42.

PERCIVAL R, URWIN GH, HARRIS S, YATES AJ, WILLIAMS JL, BENETON M AND KANIS JA. (1987). Biochemical and histological evidence that carcinoma of the prostate is associated with increased bone resorption. *Eur. J. Surg. Oncol.*, **13**, 41–49.

RISTELI J, ELOMAA I, NIEMI S, NOVAMO A AND RISTELI L. (1993). Radioimmunoassay for the pyridinoline cross-linked carboxyterminal telopeptide of type I collagen: a new serum marker of bone collagen degradation. *Clin. Chem.*, **39**, 635–640.

SANDHU DPS, MAYOR PE, SAMBROOK P AND GEORGE NJR. (1990). Increased survival of patients with massive lymphadenopathy and prostate cancer: evidence of heterogenous tumour behavior. *Br. J. Urol.*, **66**, 415–419.

SHIMAZAKI J, ISAKA S, AKIMOTO S, SIMIYA H, MASAI M AND HIGA T. (1990). Investigating the response of prostatic cancer to endocrine therapy. In *EORTC Genitourinary Group Monograph 7, Prostate Cancer and Testicular Cancer*, Newling DWW and Jones G (eds) pp. 59–67. Wiley-Liss: New York.

SOLOWAY MS, HARDEMAN SW, HICKEY D, RAYMOND J, TODD B, SOLOWAY S AND MOINUDDIN M. (1988). Stratification of patients with metastatic prostate cancer based on extent of disease on initial bone scane. *Cancer*, **61**, 195–202.

STEIN GS, LIAN JB, OWEN TA. (1990). Relationship of cell growth to the regulation of tissue-specific gene expression during osteoblast differentiation. *FASEB J.*, **4**, 3111–3123.

TAUBE T, KYLMÄLÄ T, LAMBERG-ALLARDT C, TAMMELA T AND ELOMAA I. (1994). Treatment of bone metastases from prostate cancer with estramustine phosphate and clodronate. A randomized placebo-controlled study. *Eur. J. Cancer*, (in press).

UICC (1987). *TNM Classification of Malignant Tumours*. 4th edn. pp. 124–126. Springer-Verlag.

URWIN GH, PERCIVAL RC, HARRIS S, BENETON MNC, WILLIAMS JL AND KANIS JA. (1985). Generalized increase in bone resorption in carcinoma of the prostate. *Br. J. Urol.*, **57**, 721–723.

VAES G. (1988). Cellular biology and biological mechanisms of bone resorption. *Clin. Orthop. Rel. Res.*, **231**, 239–271.